# Amyloid-β with isomerized Asp7 cytotoxicity is coupled to protein phosphorylation

**DOI:** 10.1038/s41598-018-21815-x

**Published:** 2018-02-23

**Authors:** O. G. Zatsepina, O. I. Kechko, V. A. Mitkevich, S. A. Kozin, M. M. Yurinskaya, M. G. Vinokurov, M. V. Serebryakova, A. P. Rezvykh, M. B. Evgen’ev, A. A. Makarov

**Affiliations:** 1 Engelhardt Institute of Molecular Biology, Russian Academy of Sciences, Moscow, Russia; 2 Institute of Cell Biophysics, Russian Academy of Sciences, Pushchino, Moscow Region, Russia; 3 A.N. Belozersky Institute of Physico-Chemical Biology MSU, Moscow, Russia

## Abstract

Neuronal dysfunction and loss associated with the accumulation of amyloid-β (Aβ) in the form of extracellular amyloid plaques and hyperphosphorylated tau in the form of intraneuronal neurofibrillary tangles represent key features of Alzheimer’s disease (AD). Amyloid plaques found in the brains of AD patients are predominantly composed of Aβ42 and its multiple chemically or structurally modified isoforms. Recently, we demonstrated that Aβ42 with isomerised Asp7 (isoAβ42) which is one of the most abundant Aβ isoform in plaques, exhibited high neurotoxicity in human neuronal cells. Here, we show that, in SH-SY5Y neuroblastoma cells, the administration of synthetic isoAβ42 rather than intact Aβ42 resulted in a significantly higher level of protein phosphorylation, especially the phosphorylation of tau, tubulins, and matrin 3. IsoAβ42 induced a drastic reduction of tau protein levels. Our data demonstrate, for the first time, that isoAβ42, being to date the only known synthetic Aβ species to cause AD-like amyloidogenesis in an animal AD model, induced cell death by disabling structural proteins in a manner characteristic of that observed in the neurons of AD patients. The data emphasize an important role of isoAβ42 in AD progression and provide possible neurotoxicity paths for this particular isoform.

## Introduction

Alzheimer’s disease (AD) is a progressive neurodegenerative disorder most common in ageing people and is characterized clinically by memory loss and cognitive decline^1^. The neuromorphological hallmarks of AD are cerebral amyloidogenesis, i.e., the accumulation of amyloid-β (Aβ) in the form of extracellular, insoluble aggregates (so-called amyloid plaques) in specific brain regions, intraneuronal neurofibrillary tangles (the major component of which is hyperphosphorylated tau protein), and neuronal degeneration^2^. According to a widely accepted amyloid hypothesis, the primary process triggering the pathogenesis of AD is the formation of soluble neurotoxic oligomers of Aβ^3^. Human oligomers also induce the hyperphosphorylation of tau at AD-relevant epitopes and cause neuritic dystrophy in cultured neurons^4^. It is suggested that amyloid plaques represent the major source of neurotoxic forms of Aβ oligomers^5,6^.

Intracerebral injections of homogenates from the brains of AD patients induce the whole spectrum of AD-specific disruptions in the brains of model animals^7^. Amyloid plaques from an AD brain contain a wide array of other forms of Aβ peptide (39–43 aa length) and their post-translationally modified isoforms besides the major Aβ42 peptide^8,9^. Furthermore, in various animal models of AD, chemically or structurally modified Aβ rather than intact Aβ, drastically accelerates cerebral amyloidogenesis^7^, likely due to the active involvement of physiologically intact, endogenous Aβ molecules in a chain reaction initiated by a seed-like mechanism^10^.

One of the most common component of amyloid plaques is the Aβ isoform with an isomerized aspartic acid residue at position 7 (isoAβ)^8,11^. We hypothesized that this Aβ isoform is a major player in AD pathogenesis^12,13^. We have shown that, in contrast to intact Aβ42, a synthetic peptide corresponding to isoAβ42 causes cerebral amyloidogenesis in AD animal models^14,15^. In addition, the toxic effect of isoAβ42 on neuronal cells is stronger than that of Aβ42^16,17^. Here, using SH-SY5Y neuroblastoma cells as a model for studying the effect of beta-amyloid^18–21^, we showed that isoAβ42 is more effective than Aβ42 in inducing the phosphorylation of a number of proteins, including tau, tubulins and matrin 3.

## Results

### Isomerization of Asp7 increased the apoptogenic properties of Aβ in SH-SY5Y cells

**Figure 1:**
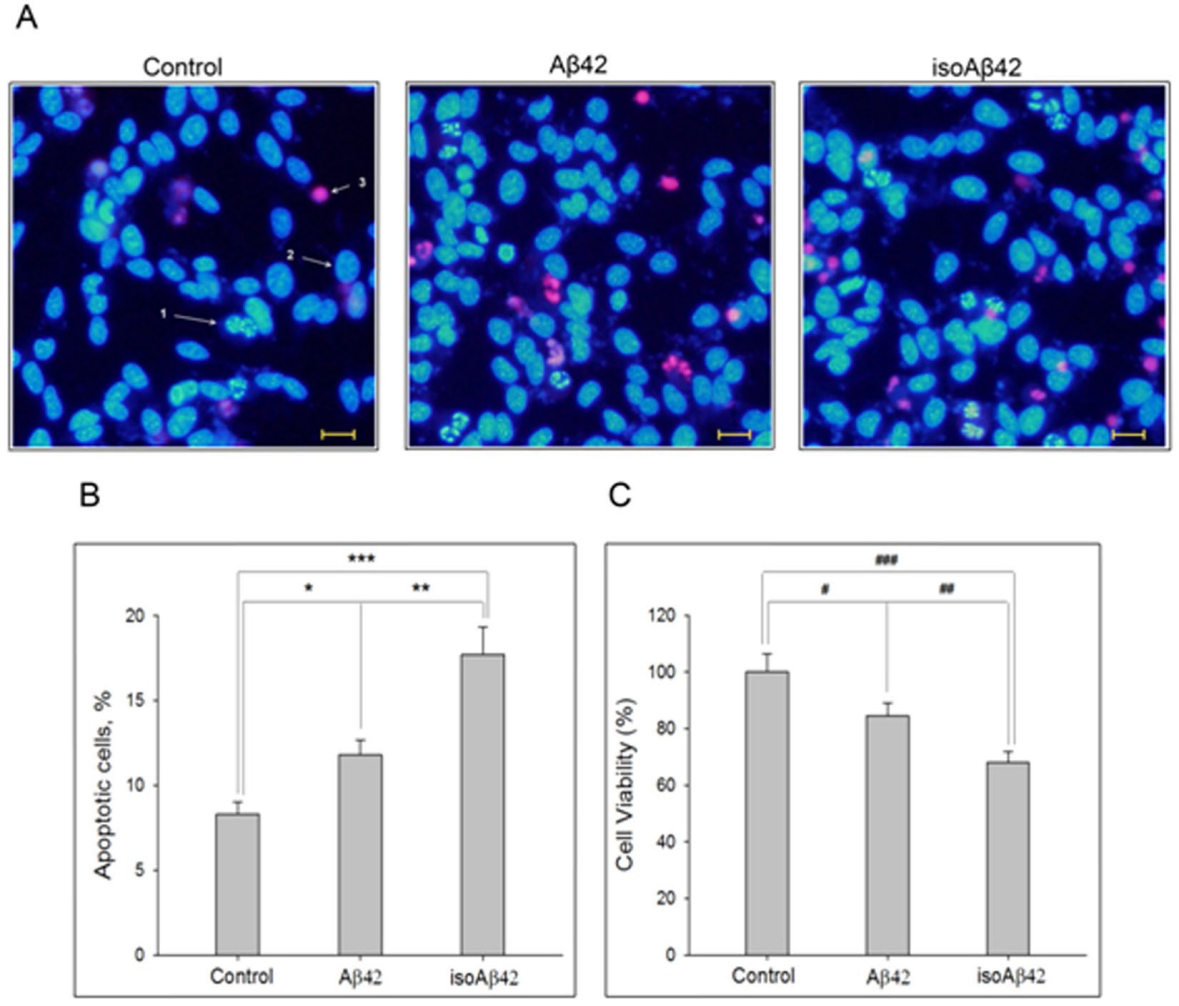
Effects of Aβ42 and isoAβ42 (10 µM) on SH-SY5Y cells. (**A**) Visualization of the cells using fluorescent microscopy. Left, untreated cells. Centre, cells treated with Aβ42. Right, cells treated with isoAβ42. Arrows indicate the following: 1 - apoptotic cells stained with Hoechst 33342; 2 - living cells; 3 - necrotic cells stained with propidium iodide. Scale is 20 µm. (**B**) Number of apoptotic cells in the cell population. Control - untreated cells; isoAβ42 and Aβ42 - cells treated with isoAβ42 and Aβ42 for 24 h, respectively. (**C**) Viability of the cells in percent, revealed by MTT-test treated with Aβ42 or isoAβ42 relative to control without treatment at 48 h. Each value represents mean ± SD of at least three independent experiments performed in quadruplicate; *p < 0.023, **p < 0.002, ***p < 0.001, ^#^p < 0.005, ^##^p < 0.003, ^###^p < 0.001.

**Figure 2:**
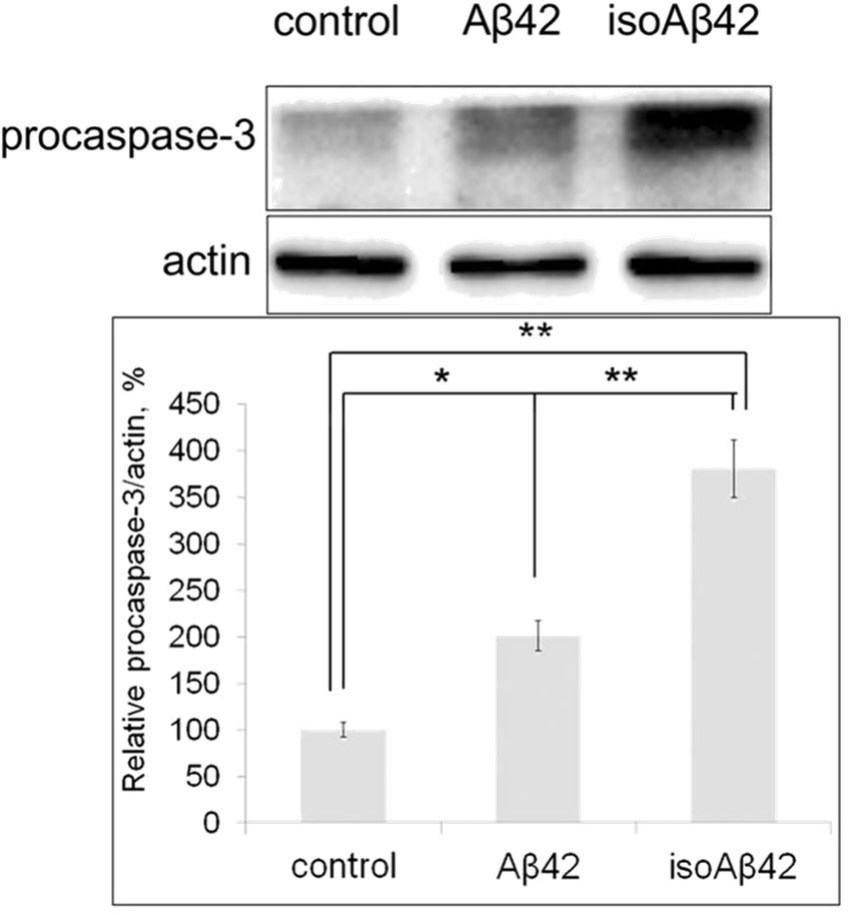
Effects of Aβ42 and isoAβ42 (10 μM, 6 h) treatment on procaspase 3 levels in SH-SY5Y cells. After peptide treatment, the cells were lysed, the isolated proteins were separated by SDS-PAGE, caspase 3 and actin were detected by Western blot using the appropriate antibodies. The bars represent procaspase 3 expression changes. Each value represents the mean ± SD of at least three independent experiments; *p < 0.02, **p < 0.005. Here and on the other figures full-length gels and blots are included in a Supplementary Information.

Both isoAβ42 and Aβ42 peptides induced SH-SY5Y cell death after 24 h of treatment; however, isoAβ42 had substantially greater toxic effects (Fig. 1). Relative to the control group of cells, the percentage of apoptotic cells in the group treated with 10 μM of isoAβ42 increased by 10%, whereas of the percentage of apoptotic cells in the group treated with Aβ42 increased by only 4% (Fig. 1B). MTT-test confirmed greater toxicity of isoAβ42 in SH-SY5Y cells in comparison with Aβ42 (Fig. 1C). Western blot analysis with antibodies to caspase-3 showed that the procaspase-3 level, an apoptosis marker, increased by two- and four-fold in SH-SY5Y cells treated with Aβ42 and isoAβ42, respectively (Fig. 2).

### Protein phosphorylation levels in SH-SY5Y cells treated with Aβ42 and isoAβ42 were different

**Figure 3:**
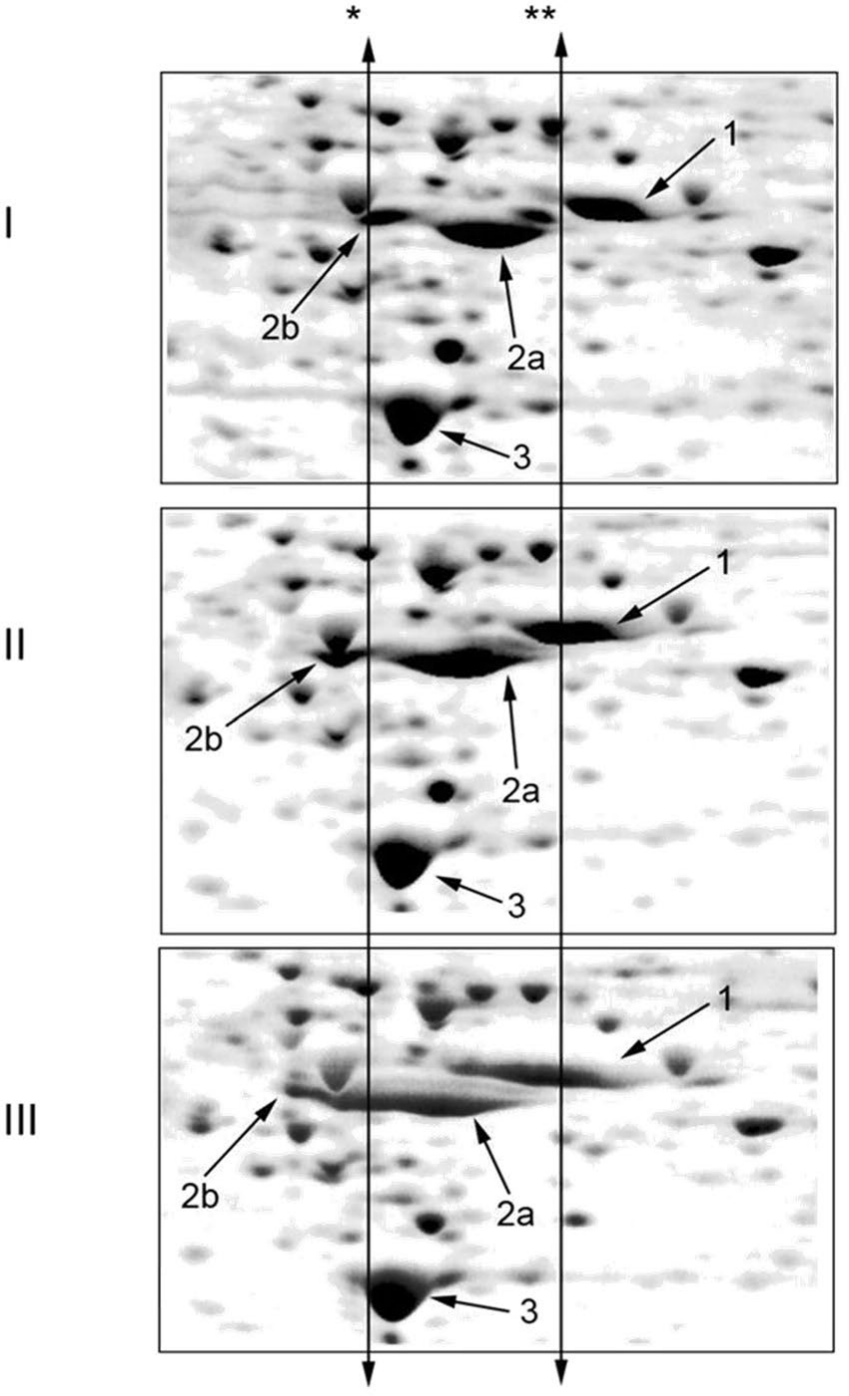
Visualization of changes in the isoelectric points of tubulin proteins after SH-SY5Y cells were treated with Aβ42 and isoAβ42 peptides (10 μM, 6 h). ^35^S-labelled proteins from control SH-SY5Y cells (**I**) and cells treated with Aβ42 (**II**) or isoAβ42 (**III**) were separated by 2D electrophoresis. The double arrow shows *the position of the acid end of β-tubulin in the control cells and ** the position of α-tubulin in the control cells. Protein identification: 1 - α-tubulin; 2a - β-tubulin (TBB5); 2b - β-tubulin (TBB4); 3-– actin (ACTB).

**Figure 4:**
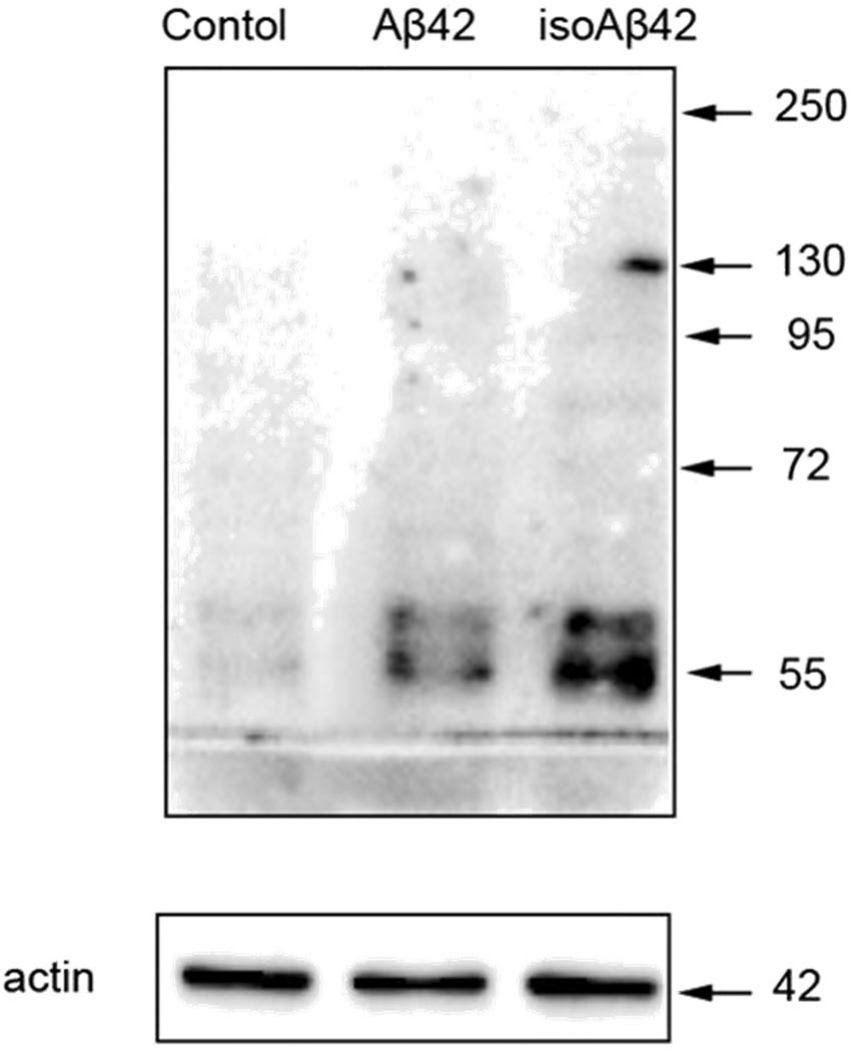
Effects of Aβ42 and isoAβ42 (10 μM, 6 h) treatment on the levels of phosphoserine-containing proteins. After incubation with the amyloid peptides, SH-SY5Y cells were lysed, the proteins were separated using 8% SDS-PAGE, and antibodies against phosphoserine were used to identify phosphoserine-containing protein bands.

**Figure 5:**
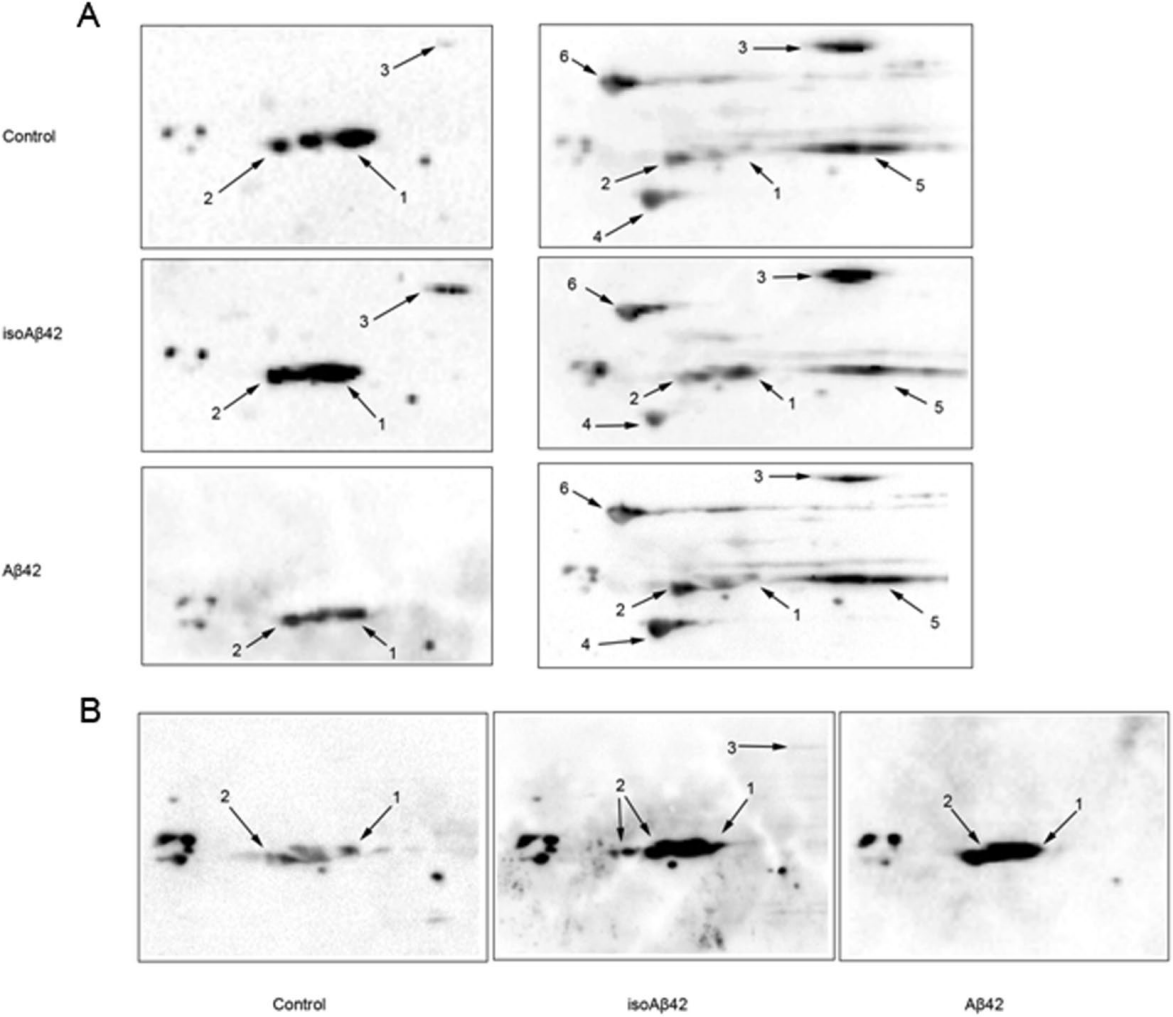
Western blot analysis of 2D-gels from SH-SY5Y control cells and SH-SY5Y cells treated with Aβ42 and isoAβ42 (10 μM, 6 h). (**A**) A merged image of anti-actin, anti-matrin 3, anti-total tau, anti-β-tubulin, anti-α-tubulin and anti-HSP90 antibodies (1 - α-tubulin, 2 - β-tubulin, 3 - matrin 3, 4 - actin, 5 - total tau, 6 – HSP90). (**B**) Anti-phosphoserine antibodies (1 - α-tubulin, 2 - β-tubulin, 3 - HNRH1, 4 - matrin 3).

**Figure 6:**
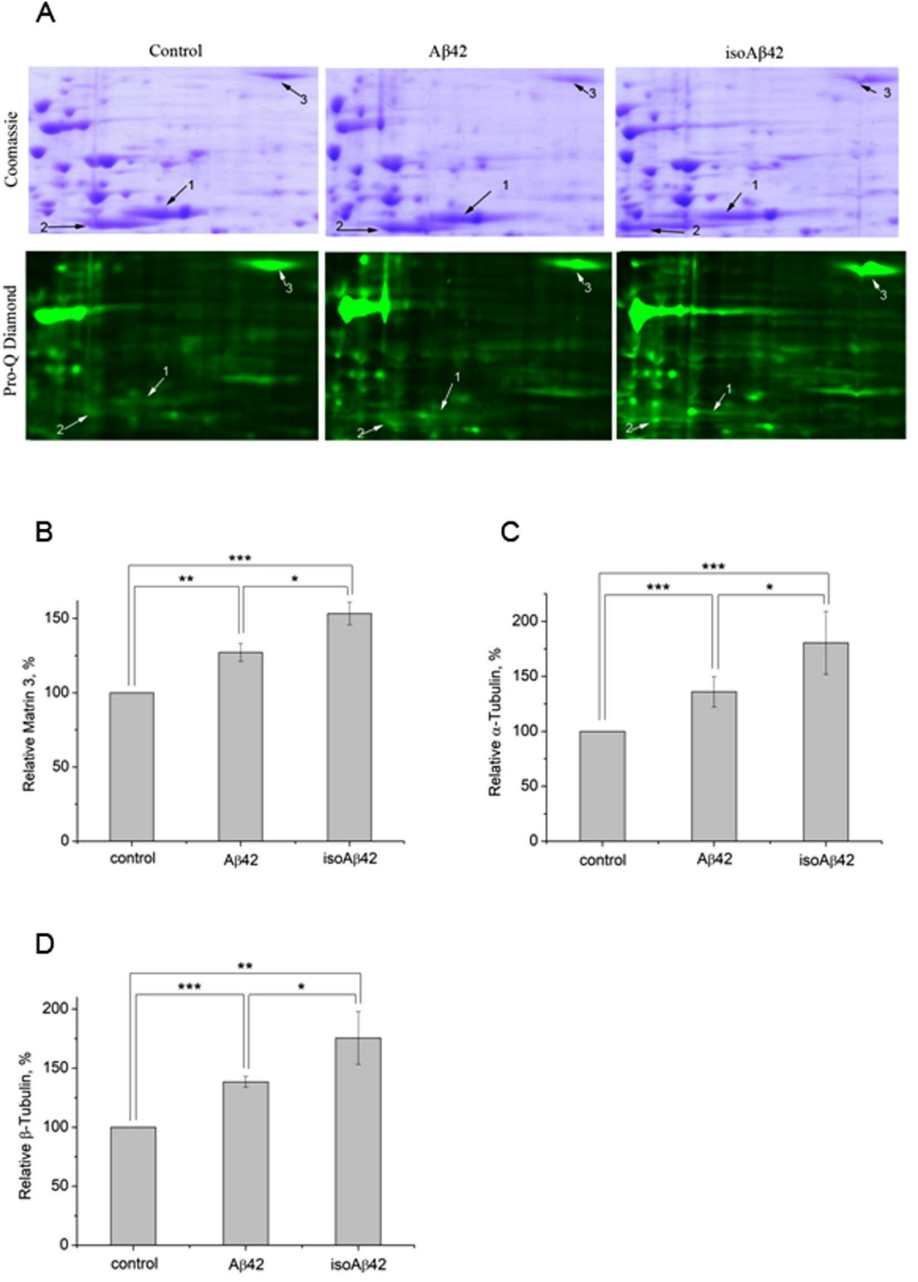
Visualization of the changes in protein phosphorylation levels after SH-SY5Y cells incubation with Aβ42 or isoAβ42 (10 μM, 6 h). Extracted proteins were separated by 2D electrophoresis. In the second dimension, proteins were separated on 11% SDS-PAGE, followed by Pro-Q diamond phosphoprotein staining and after image acquisition were restained with Comassie R -250 (**A**). 1 – α-tubulin, 2 - β-tubulin, 3- matrin 3. Densitometric analysis was performed using Fiji ImageJ software. Phosphorylated matrin 3 (**B**), α-tubulin (**C**) and β-tubulin (**D**) stained with Pro-Q diamond phosphoprotein stain were normalized to its total amount stained with Coomassie R-250. Each value is the mean expressed as a percentage of the protein phosphorylation level in the control group ± SD of at least three independent experiments; *p < 0.05, **p < 0.005, ***p < 0.001.

We performed 2D analysis of ^35^S-labeled protein lysates from control SH-SY5Y cells and cells incubated for 6 hours withAβ42 and isoAβ42 peptides. The treatment of SH-SY5Y cells with Aβ42 and isoAβ42 for 6 h changed the isoelectric points (pI) of several proteins. There was a significant shift in the pI values for α- and β-tubulin to the acid zone, which was more drastic in cells treated with isoAβ42 (Fig. 3). Because the hyperphosphorylation of β-tubulin is a hallmark of AD in the brains of patients^22^, we suggested that the observed shift was at least partially due to Aβ-induced phosphorylation. Using Western blot analysis with antibodies recognizing phosphoserine, we showed that incubation of SH-SY5Y cells with Aβ42 and isoAβ42 led to a significant increase in the phosphorylation levels of a specific set of proteins (Fig. 4); among them were both forms of tubulin (Fig. 5A and B). In contrast to Aβ42, isoAβ42 caused the appearance of additional isoforms of phosphorylated β-tubulin (Fig. 5B). IsoAβ42 treatment, but not Aβ42 treatment, caused the phosphorylation at serine residues of another vital protein, matrin 3 (Fig. 5A). To confirm the results of Western blot analysis major proteins from this area including β-tubulin, α-tubulin and matrin3 were validated by mass spectrometry followed by a search using Mascot software (Suppl. Table 1 and Suppl. Fig. 1). Additionally, gels after second dimension were stained with pro-Q diamond phosphoprotein gel stain. Phosphoprotein staining verified our conclusion that Aβ42 and especially isoAβ42 changes phosphorylation level of tubulins and matrin 3 (Fig. 6 and Suppl. Fig. 6).

### IsoAβ42 decreased tau protein levels and induced its hyperphosphorylation

**Figure 7:**
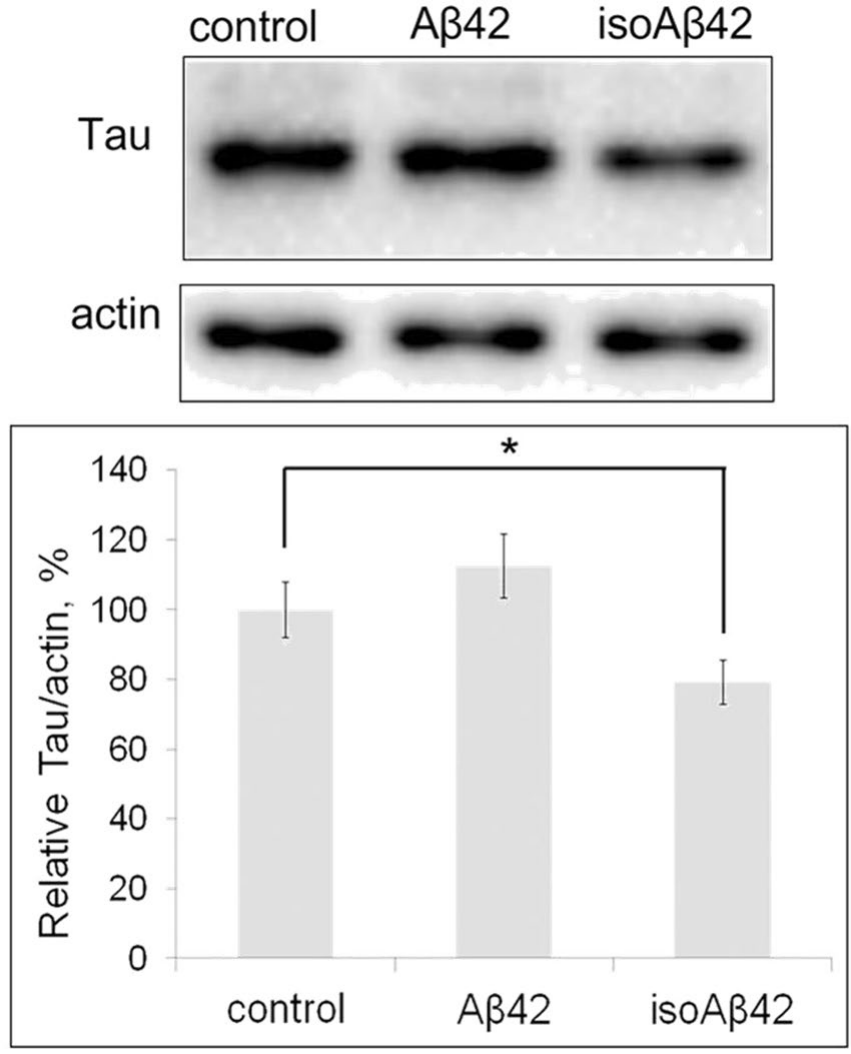
Effect of Aβ42 and isoAβ42 (10 μM, 6 h) treatment on the total tau protein level. After incubation with the amyloid peptides, the SH-SY5Y cells were lysed, the proteins in the cell lysate were separated by SDS-PAGE, and total tau was detected by Western blot using antibodies against total tau. The total tau level is expressed as a percentage of the tau level in the control group, which was not treated with Aβs. The bars represent the changes in the total tau level. Each value is the mean ± SD of at least three independent experiments; *p < 0.02.

**Figure 8:**
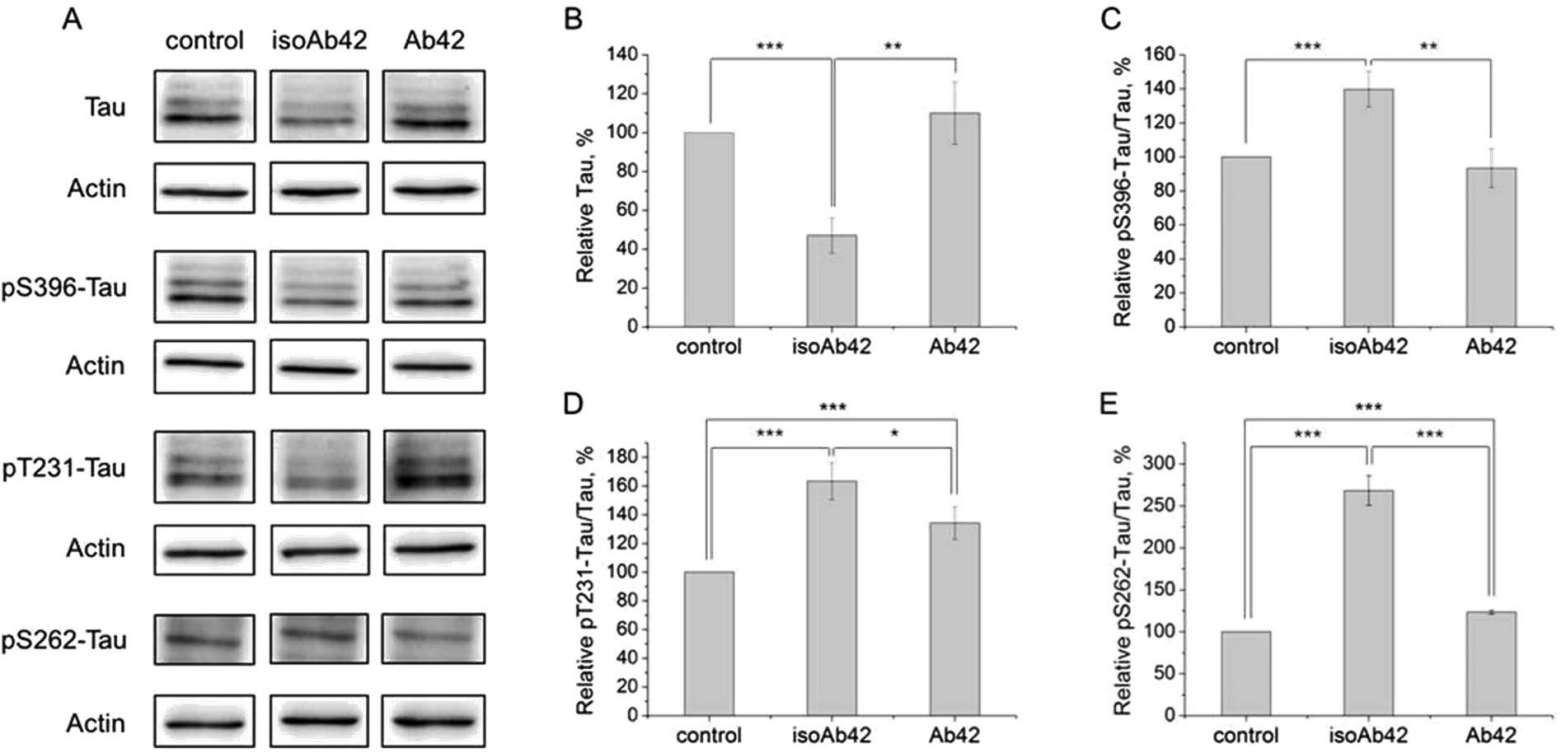
Effect of Aβ42 and isoAβ42 treatment (10 μM, 24 h) on the total tau level and the phosphorylated tau level in SH-SY5Y cells. After incubation with Aβ42 or isoAβ42, the cells were lysed, and the proteins were separated by SDS-PAGE and detected by Western blot using antibodies against total tau, phosphorylated S396 tau, phosphorylated T231 tau or phosphorylated S262 tau (**A**). The bars represent the changes in the total tau level (**B**), phosphorylated S396 tau (**C**), phosphorylated T231 tau (**D**) or phosphorylated S262 tau (**E**), expressed as a percentage of the total tau level in the control group, which was not treated with Aβ. Changes to tau phosphorylation levels were normalized to its total amount. Each value is the mean ± SD of at least three independent experiments; *p < 0.02, **p < 0.005, ***p < 0.001.

The accumulation of hyperphosphorylated tau in damaged brain regions is one of the major hallmarks of AD patients. After 6 h of treatment, neither Aβ42 nor isoAβ42 significantly influenced tau phosphorylation levels; isoAβ42 treatment decreased the amount of tau in the cells by 30% (Fig. 7). Treatment with isoAβ42 for 24 h resulted in a drastic drop (53%) of tau levels, while Aβ42 treatment did not induce any significant changes in the tau level (Fig. 8A,B). To evaluate the degree of tau phosphorylation, we used antibodies against tau phosphorylated at the S262, S396 and T231 residues. Aβ42 treatment increased the phosphorylation of tau at S262 by 23% and at T231 by 34%. IsoAβ42 treatment increased phosphorylation at S262 by 168%, at T231 by 63%, and at S396 by 40% (Fig. 8A,C–E).

## Discussion

AD includes a broad spectrum of profound molecular, histological and cognitive disturbances^2^. It is widely accepted that the oligomerization of Aβ peptides and the occurrence of intracellular neurofibrillary tangles composed of hyperphosphorylated tau protein are responsible for the observed global brain dysfunction^23^. In the normal brain, Aβ peptides are involved in synaptic activity and provide protection against excessive glutamate release^24,25^. These peptides also participate in the monitoring of cholesterol transport^26^ necessary for neuronal survival. Importantly, direct injections of synthetic Aβ42 into the brains of model animals fail to induce AD-like manifestations^7^. However, the administration of isoAβ42 induces massive amounts of amyloid plaques in the brains of AD-model mice^14^ and exerts significantly higher apoptogenic effects on model neurons and neuroblastoma cells (SK-N-SH and SH-SY5Y) than Aβ42^16,17^ (Fig. 1). These data suggest that the presence of isoAβ42 in the brain triggers the development of AD pathology. The probability of isoAβ42 formation in the human brain increases significantly with age, when the catalytic activity of protein L-isoaspartyl (D-aspartyl) methyltransferase (PIMT), responsible for isoasparagine repair is diminished^27^, which may explain the high frequency of sporadic forms of AD observed in ageing people.

Aβ42 alters the phosphorylation levels of many proteins^28^; however, isoAβ42 treatment resulted in significantly higher levels of phosphorylation of many proteins than Aβ42 treatment (Figs 4 and 6, Suppl. Fig. 6). Compared with Aβ42 treatment, isoAβ42 treatment induced higher numbers of phosphorylated isoforms of β-tubulin (Fig. 5B). In AD brains, β-tubulin is hyperphosphorylated at serine residues^22^. This event leads to the disturbance of microtubule (MT) assembly. Phosphorylated bovine brain β-tubulin loses its ability to assemble MTs and, when dephosphorylated, tubulin regains this vital ability^29^. Our experiments demonstrated that the two Aβ peptides that were used increased α-tubulin phosphorylation (Figs 5 and 6, Suppl. Fig. 6), which increases its solubility and disrupts MT assembly^30^.

Tau proteins play a major role in regulating neuronal MT assembly and stability^31,32^. Tau promotes the polymerization of tubulin into MTs^33^ and, when bound to MTs, helps stabilize them in their polymerized state^34^. The deleterious action of Aβ42 oligomers is associated with the hyperphosphorylation of tau, which results in neurofibrillar tangles and neuronal cell death^23^. We showed here that isoAβ42 administration, in contrast to Aβ42, decreased tau levels in human neuroblastoma cells, which may result in disruption of MT assembly. Normal tau levels are significantly decreased in the supernatants from the brains of AD cases^35^. However, it has not been demonstrated whether tau aggregation actually lowers the levels of soluble tau *in vivo* in AD patients^32^. IsoAβ42 treatment induced tau phosphorylation at S262, T231 and S396 residues more efficiently than Aβ42 treatment did (Fig. 8). Notably, phosphorylation of all these residues is associated with AD progression. Phosphorylation of S262 inhibits the binding of tau with MTs and concurrently activates a cascade of kinases that phosphorylate tau at other residues^36^. Furthermore, phosphorylation at T231 regulates the binding of tau with MTs and affects the stability of MT stability^37,38^, while S396 phosphorylation enhances Aβ-induced mitochondrial injury, which contributes to neuronal dysfunction and to the pathogenesis of AD^39^. It is evident that the observed alterations in the phosphorylation of tubulins and tau induced by isoAβ42 should result in severe disturbances in neurotransmission, MT formation and eventually neuronal death. The hyperphosphorylation of matrin 3 at serine residues (Fig. 5), observed after isoAβ42 treatment but not after Aβ42 treatment, may be another factor responsible for the higher level of neuronal death detected with isoAβ42 treatment (Fig. 1). Matrin 3 is a highly conserved nuclear matrix phosphoprotein implicated in transcription and interaction with other nuclear matrix protein to form the internal fibrogranular network^40,41^. Hyperphosphorylation of matrin 3 in neurons leads to their degradation^42^.

In summary, we have shown, for the first time, that the significantly higher cytotoxic effect of isoAβ42 than that of intact Aβ42 is associated with the role of isoAβ42 as the inducer of more effective phosphorylation of several structural proteins including tau, the tubulins, and matrin 3. Hyperphosphorylation of these proteins is a hallmark of AD brains and is linked to neuronal death, which suggests a triggering role for isoAβ42 in this pathological cascade.

## Materials and Methods

### Cells and peptides

The SH-SY5Y neuroblastoma cell line was obtained from the European Collection of Authenticated Cell Culture (ECACC, Public Health England, UK). Cells were cultured in DMEM (Sigma Aldrich) supplemented with 10% heat-inactivated defined foetal calf serum (FCS, HyClone), 2 mM L-glutamine, 100 units/ml of penicillin and 100 μg/ml streptomycin at 37 °C in a humid atmosphere with 5% CO_2_. The synthetic peptides, isoAβ42 and Aβ42, were purchased from Biopeptide and prepared as described previously^16^. Cold hexafluoroisopropanol (Fluka) was added to dry Aβ42 or isoAβ42 to a concentration of 1 mM and incubated for 60 min at room temperature. Then this solution was put on ice for 10 min and aliquoted into non-siliconized microcentrifuge tubes (0.56 mg peptide per tube). Peptide in the tubes was dried under vacuum using Eppendorf Concentrator 5301. Dried peptide was stored at −80 °C. For addition to cells, 2.5 mM peptide stock solution was prepared by adding 20 µl of 100% anhydrous DMSO (Sigma-Aldrich) to 0.22 mg peptide and incubating for 1 h at room temperature. For use in the experiments, the peptide was diluted to the required concentration with buffer solution. Equivalent amount of DMSO was added to the control samples in all experiments. Based on our previous data for cell treatment we used peptides in concentration 10 µM which is optimal for *in vitro* studies of amyloid pathogenic effects on neuroblastoma cells^16,17,43^.

### Cell viability assay

Cell viability was assessed with MTT-test kit (Sigma). Briefly, SH-SY5Y cells were seeded in 96-well plates and cultured for 24 h at 37 °C. Then, the cells were treated with isoAβ42 or Aβ42 for 48 h followed by incubation with MTT reagent for 4 h at 37 °C. The absorbance of samples was measured in a multiscan FC microplate reader (Thermo Fisher Scientific) at 570 nm. Viability of untreated cells was taken as 100%.

### Apoptosis assay by Hoechst 33342 staining

To study apoptosis, cells were collected using trypsin-versene, washed with complete culture medium, counted, and seeded into four-well plates (Nunc, Thermo Fisher Scientific, USA), coated with 0.01% Poly-L-lysine, at 400,000 cells per well in 1 mL of culture medium supplemented with 5% FCS and were cultivated at 37 °C in a humid atmosphere with 5% CO_2_ for 24 h. After 24 h, the medium with 5% FCS was substituted with FCS-free medium. IsoAβ42 or Aβ42 peptides were added to the wells, and the cells were cultured for 24 h at 37°С. Apoptotic cells were detected using fluorescent microscopy and Hoechst 33342 dye^44^, and necrotic cells were detected using propidium iodide. After the culture medium was washed off, the cell layer was stained with 10 μg/ml Hoechst 33342 dye in phosphate buffer for 30 min at 37 °С in the dark. Then, 30 μM propidium iodide was added, and the cells were visualized using an inverted fluorescent microscope (Keyence BZ8100, Japan). The number of apoptotic cells was calculated as a portion of the number of cells with fragmented DNA (not stained with propidium iodide) out of the total number of cells (100%). Cells that were stained with propidium iodide were considered necrotic. To register apoptosis, at least 20 fields of view were analysed, each containing 250–350 cells. Results of three experiments with four repetitions were pooled and statistically processed.

### Probe preparation for 2D analysis

To obtain samples for 2D electrophoresis, SH-SY5Y cells were harvested from culture flasks using a trypsin-EDTA solution (Sigma-Aldrich) and washed twice with culture medium. The pellet was resuspended, and the cells were counted, diluted in serum-free culture medium, and transferred into glass tubes. Then, the cells were treated with either Aβ42 (10 μM) or isoAβ42 (10 μM) for 6 or 24 h at 37 °С and 5% СО_2_. Following the incubation, the cells were placed in an ice bath for 15 min, centrifuged, and washed three times in Hanks’ Balanced Salt solution (HBSS, Sigma-Aldrich). The cell pellets were resuspended in HBSS and 2.0 × 10^6^ cells were labelled with 1.85 MBq of L-[^35^S]methionine (Amersham Biosciences Corp., Piscataway, NJ, USA) in HBSS for 1 h at 37 °C. An unlabelled sample was prepared at the same time. The 2 × 10^6^ L-[^35^S]methionine-labelled and unlabelled cells were lysed by stirring at 4 °C for 15 min in 70 µl of O’Farrell lysis buffer with 1% PMSF and 1% protease-inhibitor cocktail (Amresco). Halt Phosphatase Inhibitor Cocktail (Thermo scientific) was added into the samples for staining with pro-Q diamond phosphoprotein gel stain.The probes were then centrifuged at 15,000 g for 10 min, and the supernatant was collected.

### 2D electrophoresis and data analysis

2D PAGE was performed by electrofocusing in a polyacrylamide gel at a pH range of approximately 4.5 to 9.5 in the first direction followed by SDS-11% PAGE in the second direction using a modified version of O’Farrell’s method^45,46^. After electrophoresis, the gels were stained with a 0.2% solution of Coomassie Brilliant Blue G250 (CBB), destained, and dried for viewing using the Typhoon FLA 9500 imaging system from GE Healthcare Life Sciences. To reveal phosphorylated proteins, gels after second direction were stained with pro-Q diamond phosphoprotein gel stain (Molecular Probes, Invitrogen, UK) according to manufacturer’s instructions and viewed on Bio-Rad ChemiDoc MP gel imaging system. After imaging, gels were stained with a 0.2% solution of Coomassie Brilliant Blue G250. Densitometric analysis was performed using Fiji ImageJ software.

### MALDI-TOF mass spectrometry

Mass spectra of the tryptic peptides of from SH-SY5Y cellular proteins were obtained using a matrix-assisted laser desorption/ionization (MALDI) time-of-flight mass (TOF) spectrometer, Ultraflextreme BRUKER (Germany), equipped with a UV laser (Nd) and reflectron at the Human Proteome Shared Facility Centre at the Institute of Biomedical Chemistry (Moscow, Russia). Pieces (2 × 2 mm) of the proteins of interest from 2D-gels were analysed as described previously^47^. Identification of SH-SY5Y cellular proteins was performed using Mascot software (www.matrixscience.com) and the NCBI database, taking into account the possible oxidation of methionine residues and the modification of cysteine residues by acrylamide.

### Analysis of proteins by Western blot

For immunoblot analysis of tau phosphorylation, cells were incubated with 10 μM Aβ42 or isoAβ42 for 24 h and then lysed by stirring at 4 °C for 1 h in RIPA buffer (25 mM Tris-HCl, pH 7.6, 150 mМ NaCl, 1% Nonidet-P40, 0.1% SDS, 1% sodium deoxycholate), containing 200 μM PMSF and a complete protease-inhibitor mixture (Roche). The lysate and probes were then centrifuged at 15,000 g for 10 min, and the supernatant was collected. Proteins from the cell lysates and proteins isolated after the first gel electrophoresis on a 2D gel electrophoresis were separated by SDS-PAGE and transferred to a nitrocellulose membrane. The membranes were stained with Ponceau S (Sigma, USA) to monitor transfer efficiency. After the membranes were blocked with 5% nonfat milk or 5% BSA in PBST, primary antibodies (Suppl. Table 2) were used to detect the proteins. Then, the blots were incubated with the appropriate secondary antibodies (Suppl. Table 2). Protein visualization was performed using the appropriate horseradish peroxidase-conjugated secondary antibodies provided by the enhanced chemiluminescence SuperSignal™ West Femto Maximum Sensitivity Substrate kit (Thermo Scientific). Chemiluminescence was detected using a Bio-Rad ChemiDoc MP gel imaging system. Densitometric analysis was performed using Image Lab software (Bio-Rad), and the results are expressed as the ratio of phospho-tau band density to total tau band intensity.

### Statistical analyses

The data are presented as the mean ± standard deviation of at least three independent experiments. The differences between the groups were analysed using One Way ANOVA with Tukey’s pairwise comparisons and p < 0.05 was considered significant.

### Data availability statement

The datasets generated during and/or analysed during the current study are available from the corresponding author on reasonable request.

## Electronic supplementary material

Supplementary materials

## References

[1] Khachaturian Z (1985). Diagnosis of Alzheimer’s disease. Arch Neurol.

[2] Cummings JLA’sD (2004). New England Journal of Medicine.

[3] Hardy J (2009). The amyloid hypothesis for Alzheimer’s disease: a critical reappraisal. Journal of Neurochemistry.

[4] Selkoe DJ, Hardy J (2016). The amyloid hypothesis of Alzheimer’s disease at 25 years. EMBO Molecular Medicine.

[5] Cohen, S. I. A. *et al*. Proliferation of amyloid-β42 aggregates occurs through a secondary nucleation mechanism. *Proceedings of the National Academy of Sciences*, 10.1073/pnas.1218402110 (2013). 10.1073/pnas.1218402110 PMC3683769 23703910

[6] Koffie RM (2009). Oligomeric amyloid beta associates with postsynaptic densities and correlates with excitatory synapse loss near senile plaques. Proc Natl Acad Sci USA.

[7] Meyer-Luehmann M (2006). Exogenous induction of cerebral beta-amyloidogenesis is governed by agent and host. Science.

[8] Roher AE (1993). Structural alterations in the peptide backbone of beta-amyloid core protein may account for its deposition and stability in Alzheimer’s disease. J Biol Chem.

[9] Roher AE (1993). beta-Amyloid-(1-42) is a major component of cerebrovascular amyloid deposits: implications for the pathology of Alzheimer disease. Proc Natl Acad Sci USA.

[10] Jucker M, Walker LC (2013). Self-propagation of pathogenic protein aggregates in neurodegenerative diseases. Nature.

[11] Hosoda R (1998). Quantification of modified amyloid beta peptides in Alzheimer disease and Down syndrome brains. Journal of neuropathology and experimental neurology.

[12] Kozin SA, Mitkevich VA, Makarov AA (2016). Amyloid-β containing isoaspartate 7 as potential biomarker and drug target in Alzheimer’s disease. Mendeleev Communications.

[13] Barykin EP, Mitkevich VA, Kozin SA, Makarov AA (2017). Amyloid beta Modification: A Key to the Sporadic Alzheimer’s Disease?. Frontiers in genetics.

[14] Kozin SA (2013). Peripherally applied synthetic peptide isoAsp7-Aβ(1-42) triggers cerebral β-amyloidosis. Neurotox Res.

[15] Kulikova AA (2016). Intracerebral Injection of Metal-Binding Domain of Abeta Comprising the Isomerized Asp7 Increases the Amyloid Burden in Transgenic Mice. Neurotoxicity research.

[16] Mitkevich VA (2013). *Isomerization of Asp7 l*eads to increased toxic effect of amyloid-β42 on human neuronal cells. Cell death & disease.

[17] Yurinskaya MM (2015). *HSP70 protects human ne*uroblastoma cells from apoptosis and oxidative stress induced by amyloid peptide isoAsp7-Abeta(1-42). Cell death & disease.

[18] Abramova NA, Cassarino DS, Khan SM, Painter TW, Bennett JP (2002). Inhibition by R(+) or S(−) pramipexole of caspase activation and cell death induced by methylpyridinium ion or beta amyloid peptide in SH-SY5Y neuroblastoma. Journal of neuroscience research.

[19] Misonou H, Morishima-Kawashima M, Ihara Y (2000). Oxidative stress induces intracellular accumulation of amyloid beta-protein (Abeta) in human neuroblastoma cells. Biochemistry.

[20] Peraus GC, Masters CL, Beyreuther K (1997). Late compartments of amyloid precursor protein transport in SY5Y cells are involved in beta-amyloid secretion. The Journal of neuroscience: the official journal of the Society for Neuroscience.

[21] Shen Y (1998). Induced expression of neuronal membrane attack complex and cell death by Alzheimer’s beta-amyloid peptide. Brain research.

[22] Vijayan S, El-Akkad E, Grundke-Iqbal I, Iqbal K (2001). A pool of beta-tubulin is hyperphosphorylated at serine residues in Alzheimer disease brain. FEBS letters.

[23] Hardy J (2014). Pathways to Alzheimer’s disease. Journal of internal medicine.

[24] Kamenetz F (2003). APP processing and synaptic function. Neuron.

[25] Lesne S, Kotilinek L (2005). Amyloid plaques and amyloid-beta oligomers: an ongoing debate. The Journal of neuroscience: the official journal of the Society for Neuroscience.

[26] Igbavboa U, Sun GY, Weisman GA, He Y, Wood WG (2009). Amyloid beta-protein stimulates trafficking of cholesterol and caveolin-1 from the plasma membrane to the Golgi complex in mouse primary astrocytes. Neuroscience.

[27] Desrosiers RR, Fanelus I (2011). Damaged proteins bearing L-isoaspartyl residues and aging: a dynamic equilibrium between generation of isomerized forms and repair by PIMT. Current aging science.

[28] Henriques AG (2016). Altered protein phosphorylation as a resource for potential ADbiomarkers. Scientific reports.

[29] Wandosell F, Serrano L, Hernandez MA, Avila J (1986). Phosphorylation of tubulin by a calmodulin-dependent protein kinase. J Biol Chem.

[30] Ley SC (1994). Tyrosine phosphorylation of alpha tubulin in human T lymphocytes. European journal of immunology.

[31] Ferreira A, Busciglio J, Caceres A (1989). Microtubule formation and neurite growth in cerebellar macroneurons which develop *in vitro*: evidence for the involvement of the microtubule-associated proteins, MAP-1a, HMW-MAP2 and Tau. Brain research. Developmental brain research.

[32] Morris M, Maeda S, Vossel K, Mucke L (2011). The many faces of tau. Neuron.

[33] Weingarten MD, Lockwood AH, Hwo SY, Kirschner MW (1975). A protein factor essential for microtubule assembly. Proc Natl Acad Sci USA.

[34] Drechsel DN, Hyman AA, Cobb MH, Kirschner MW (1992). Modulation of the dynamic instability of tubulin assembly by the microtubule-associated protein tau. Molecular Biology of the Cell.

[35] Khatoon S, Grundke-Iqbal I, Iqbal K (1994). Levels of normal and abnormally phosphorylated tau in different cellular and regional compartments of Alzheimer disease and control brains. FEBS letters.

[36] Mairet-Coello G (2013). The CAMKK2-AMPK kinase pathway mediates the synaptotoxic effects of Abeta oligomers through Tau phosphorylation. Neuron.

[37] Cho JH, Johnson GV (2004). Primed phosphorylation of tau at Thr231 by glycogen synthase kinase 3beta (GSK3beta) plays a critical role in regulating tau’s ability to bind and stabilize microtubules. Journal of neurochemistry.

[38] Schwalbe M (2015). Structural Impact of Tau Phosphorylation at Threonine 231. Structure (London, England: 1993).

[39] Quintanilla RA, von Bernhardi R, Godoy JA, Inestrosa NC, Johnson GV (2014). Phosphorylated tau potentiates Abeta-induced mitochondrial damage in mature neurons. Neurobiology of disease.

[40] Belgrader P, Dey R, Berezney R (1991). Molecular cloning of matrin 3. A 125-kilodalton protein of the nuclear matrix contains an extensive acidic domain. J Biol Chem.

[41] Coelho MB (2015). Nuclear matrix protein Matrin3 regulates alternative splicing and forms overlapping regulatory networks with PTB. The EMBO journal.

[42] Giordano G (2005). Activation of NMDA receptors induces protein kinase A-mediated phosphorylation and degradation of matrin 3. Blocking these effects prevents NMDA-induced neuronal death. Journal of neurochemistry.

[43] Barykin EP, Petrushanko IY, Burnysheva KM, Makarov AA, Mitkevich VA (2016). Isomerization of Asp7 increases the toxic effects of amyloid beta and its phosphorylated form in SH-SY5Y neuroblastoma cells. Mol Biol (Moscow).

[44] Peng Y, Hu Y, Feng N, Wang L, Wang X (2011). L-3-n-butyl-phthalide alleviates hydrogen peroxide-induced apoptosis by PKC pathway in human neuroblastoma SK-N-SH cells. Naunyn-Schmiedeberg’s archives of pharmacology.

[45] O’Farrell PZ, Goodman HM, O’Farrell PH (1977). High resolution two-dimensional electrophoresis of basic as well as acidic proteins. Cell.

[46] Bedulina D (2017). Intersexual differences of heat shock response between two amphipods (Eulimnogammarus verrucosus and Eulimnogammarus cyaneus) in Lake Baikal. PeerJ.

[47] Lyupina YV (2016). Proteomics of the 26S proteasome in Spodoptera frugiperda cells infected with the nucleopolyhedrovirus, AcMNPV. Biochimica et biophysica acta.

