# Supplementary material for: Amyloid-β with isomerized Asp7 cytotoxicity is coupled to protein phosphorylation
Source: Sci Rep. 2018 Feb 23;8:3518. doi: 10.1038/s41598-018-21815-x (PMC5824883; doi:10.1038/s41598-018-21815-x)

Supplementary materials

**Amyloid-β with isomerized Asp7 cytotoxicity is coupled to protein phosphorylation**

Zatsepina O.^1^, Kechko O.^1^, Mitkevich V.A.^1^, Kozin S.A.^1^, Yurinskaya M.^1,2^, Vinokurov M.^2^, Serebryakova M.^3^, Rezvykx A.^1^, Evgen’ev M.B.^1^, Makarov A.A.^1^

^1^Engelhardt Institute of Molecular Biology, Russian Academy of Sciences, Moscow, Russia

^2^Institute of Cell Biophysics, Russian Academy of Sciences, Pushchino, Moscow Region, Russia

^3^A.N. Belozersky Institute of Physico-Chemical Biology MSU, Moscow, Russia

Corresponding author: A.A. Makarov, Engelhardt Institute of Molecular Biology, RAS, Vavilov Street 32, Moscow, 119991, Russia; Tel: +7(499)1352311; Fax: +7(495)1351405; e-mail: aamakarov@eimb.ru

Suppl. Table 1 –Proteins identified in SH-SY5Y cells by MALDI-TOF mass spectrometry

| Protein (Human) | Full protein  name | Uniprot Entry | mass,kDa | score | pI | p-value | coverage, % | Main function | Short Reference |
| --- | --- | --- | --- | --- | --- | --- | --- | --- | --- |
| HS90A | Heat shock protein HSP 90-alpha | P08238 | 83,21 | 216 | 4,97 | 2,14E-05 | 42 | Chaperone | Molecular chaperone that promotes the maturation and proper regulation of specific target proteins involved in cell cycle control and signal transduction. |
| HSPA1 | Heat shock 70 kDa protein 1 | P0DMV8 | 70 | 289 | 5,48 | 1,20E-05 | 41 | Chaperone | Molecular chaperone implicated in protection of the proteome from stress, folding and transport of newly synthesized polypeptides. |
| HSP7C | Heat shock cognate 71 kDa protein | P11142 | 70,85 | 297 | 5,37 | 1,13E-05 | 42 | Chaperone | Molecular chaperone implicated in a wide variety of cellular processes. |
| CH60 | 60 kDa heat shock protein, mitochondrial | P10809 | 61,01 | 263 | 5,7 | 1,45E-05 | 50 | Chaperonin | Chaperonin, together with Hsp10, facilitates the correct folding of imported proteins. |
| VIME | Vimentin | P08670 | 53,62 | 426 | 5,06 | 5,51E-06 | 66 | Structural protein | Vimentins are intermediate filaments found in non-epithelial cells, Vimentin is attached to the nucleus, endoplasmic reticulum, and mitochondria. |
| ACTB | Actin, cytoplasmic 1 | P60709 | 41,71 | 99 | 5,29 | 1,02E-04 | 25 | Structural protein | Actins conserved proteins that are involved in various types of cell motility and are ubiquitously expressed in all eukaryotic cells. |
| HNRH1 | Heterogeneous nuclear ribonucleoprotein H | P31943 | 49,2 | 134 | 5,89 | 5,57E-05 | 41 | Transcription | Is a component of the heterogeneous nuclear ribonucleoprotein (hnRNP) complexes which provide processing events that pre-mRNAs undergo before becoming functional, mRNAs in the cytoplasm. |
| MATR3 | Matrin-3 | P43243 | 94,57 | 64 | 5,87 | 2,44E-04 | 13 | Transcription | May play a role in transcription or may interact with other nuclear matrix proteins to form the internal fibrogranular network. |
| EF2 | Elongation factor 2 | P13639 | 95,338 | 251 | 6,41 | 4,34E-06 | 29 | Translation | Catalyzes the GTP-dependent ribosomal translocation step during translation elongation |

Suppl. Table 2 – Antibodies used in Western blot analysis

| Antibody | Host | Manufacturer | Dilution |
| --- | --- | --- | --- |
| *Primary* |  |  |  |
| Anti-Cleaved caspase3 | Rabbit polyclonal | Cell signaling USA | 1:1000 |
| Anti-Matrin-3 | Rabbit polyclonal | Novus, USA | 1:1000 |
|  |  |  |  |
| Anti-Beta-Tubulin | Rabbit polyclonal | Cell signaling USA | 1:1000 |
| Anti-α-Tubulin (11H10) | Rabbit monoclonal | Cell signaling USA | 1:1000 |
| Anti-Hsp90 16F1 | Rat monoclonal | Enzo, USA | 1:1000 |
|  |  |  |  |
| Anti-Tau [E178] | Rabbit monoclonal | Abcam, UK | 1:5000 |
| Anti-Tau (phospho S262) | Rabbit polyclonal | Abcam, UK | 1:500 |
| Anti-Tau (phospho S396) | Rabbit monoclonal | Abcam, UK | 1:10000 |
| Anti-Tau (phospho T231) | Rabbit monoclonal | Abcam, UK | 1:1000 |
| Anti-β-actin | Mouse monoclonal | Ambion, USA | 1:20000 |
| *Secondary* |  |  |  |
| Anti-mouse HRP | Goat polyclonal | Enzo, USA | 1:10000 |
| Anti-Rat HRP | Goat polyclonal | Enzo, USA | 1:10000 |
| Anti-rabbit HRP | Goat polyclonal | Abcam, UK | 1:20000 |
| Anti-Rabbit HRP | Donkey polyclonal | Novex, USA | 1:10000 |
| Anti-Mouse HRP | Rabbit polyclonal | Imtek, RF | 1:10000 |


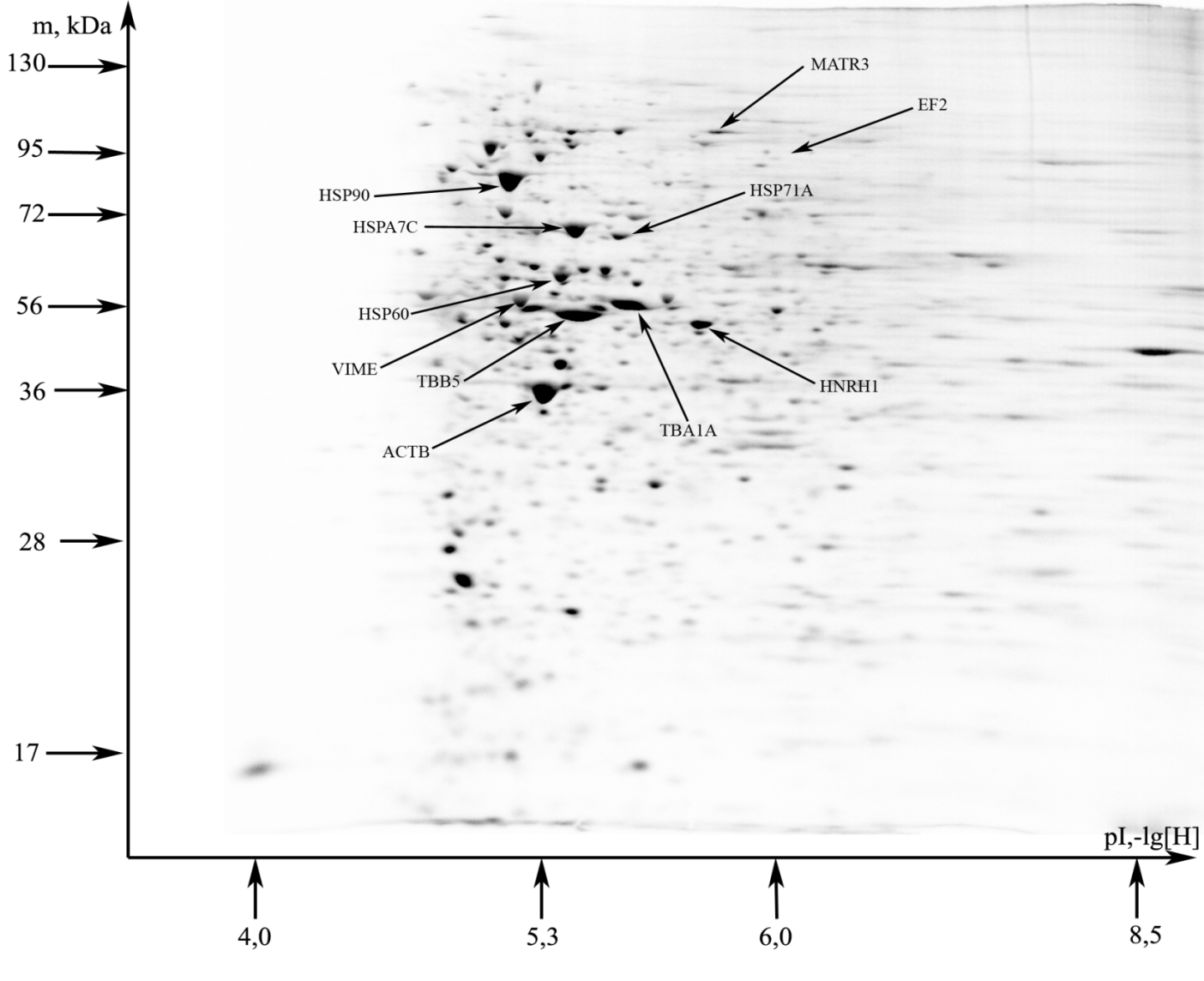


Suppl. Fig.1. 2D electrophoresis or of ^35^S labeled proteins. Protein map of identified spots in SH-SY5Y cells.

Western blot corresponding to Figure 2


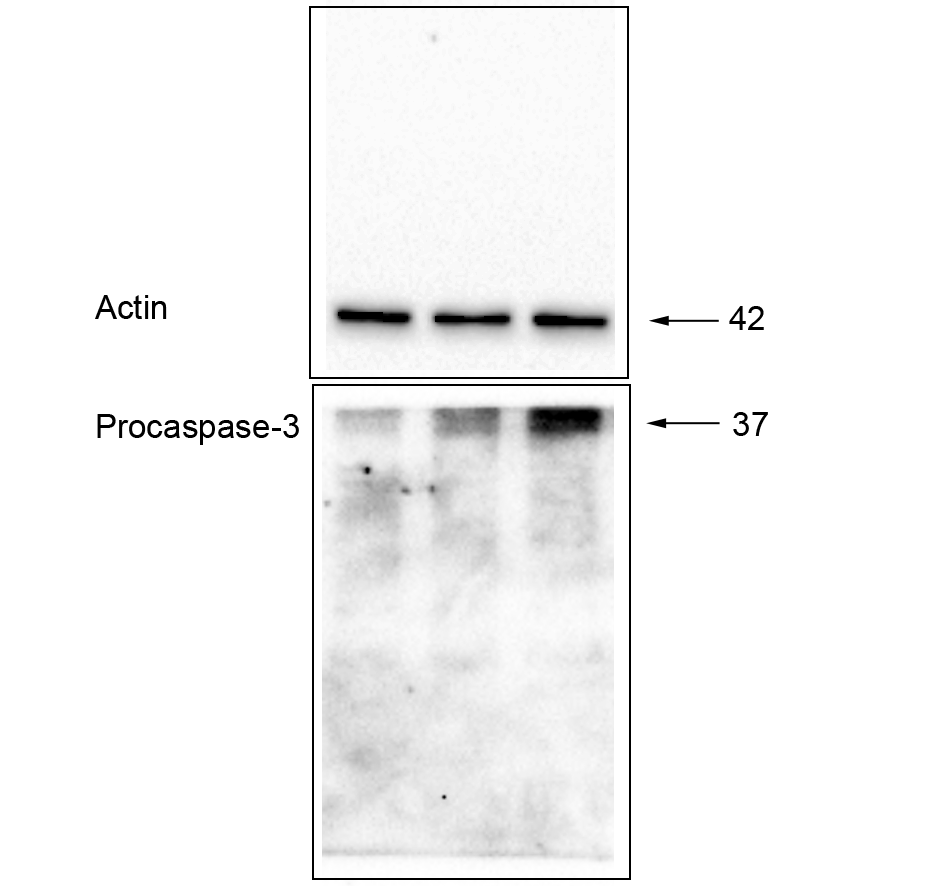


Full pictures for Figure 3, ^35^S-labelled proteins from control SH-SY5Y cells (I) and cells treated with Aβ42 (II) or isoAβ42 (III)


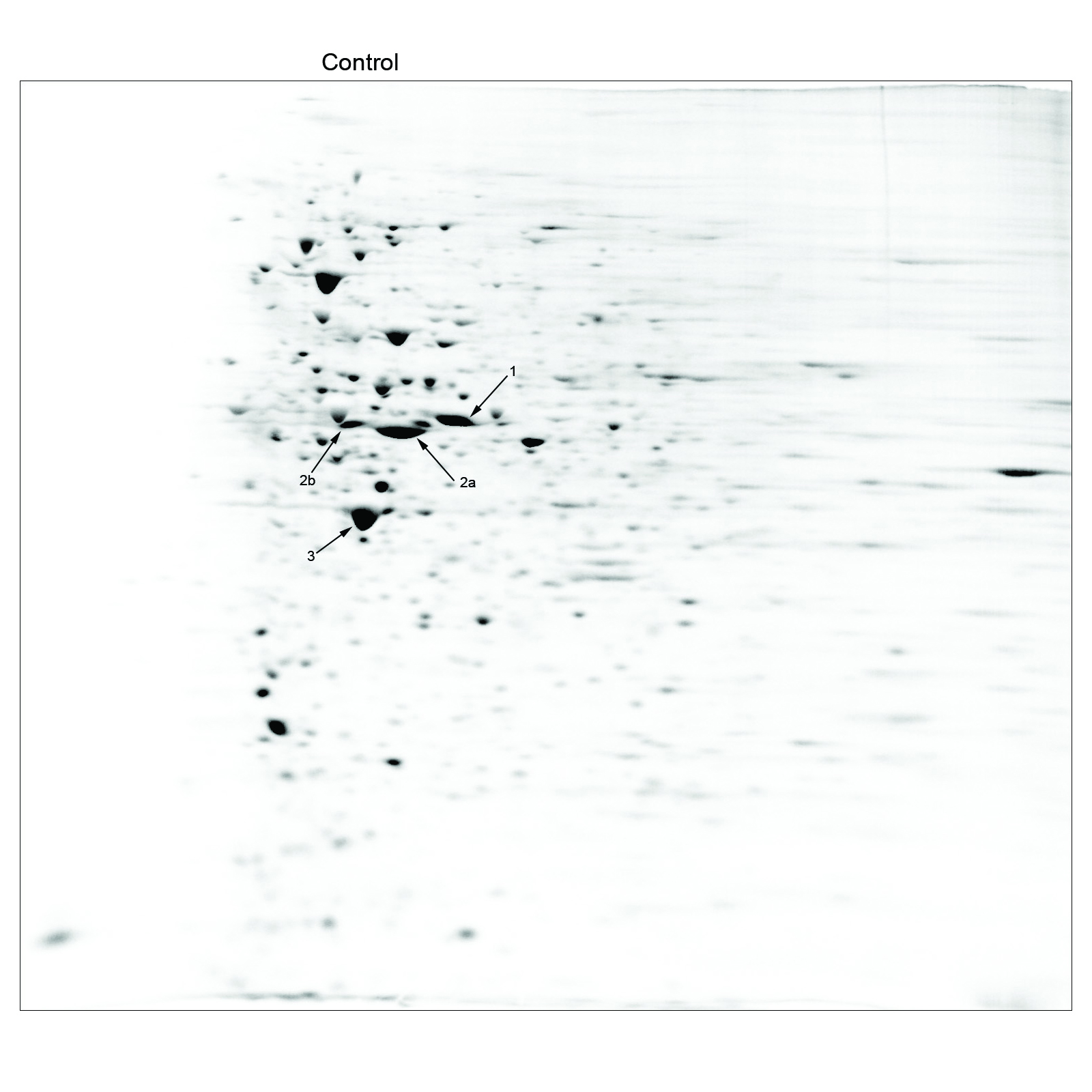

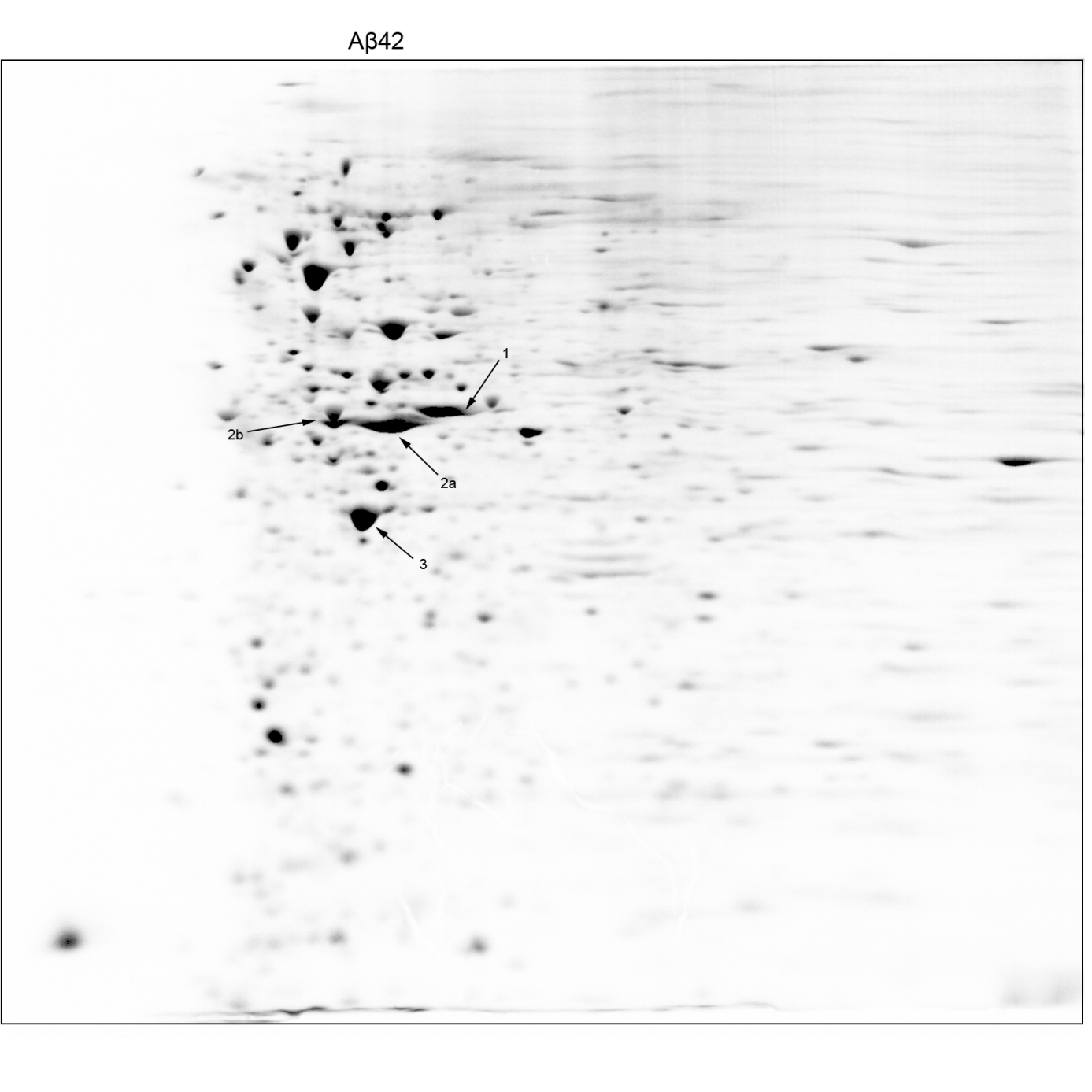


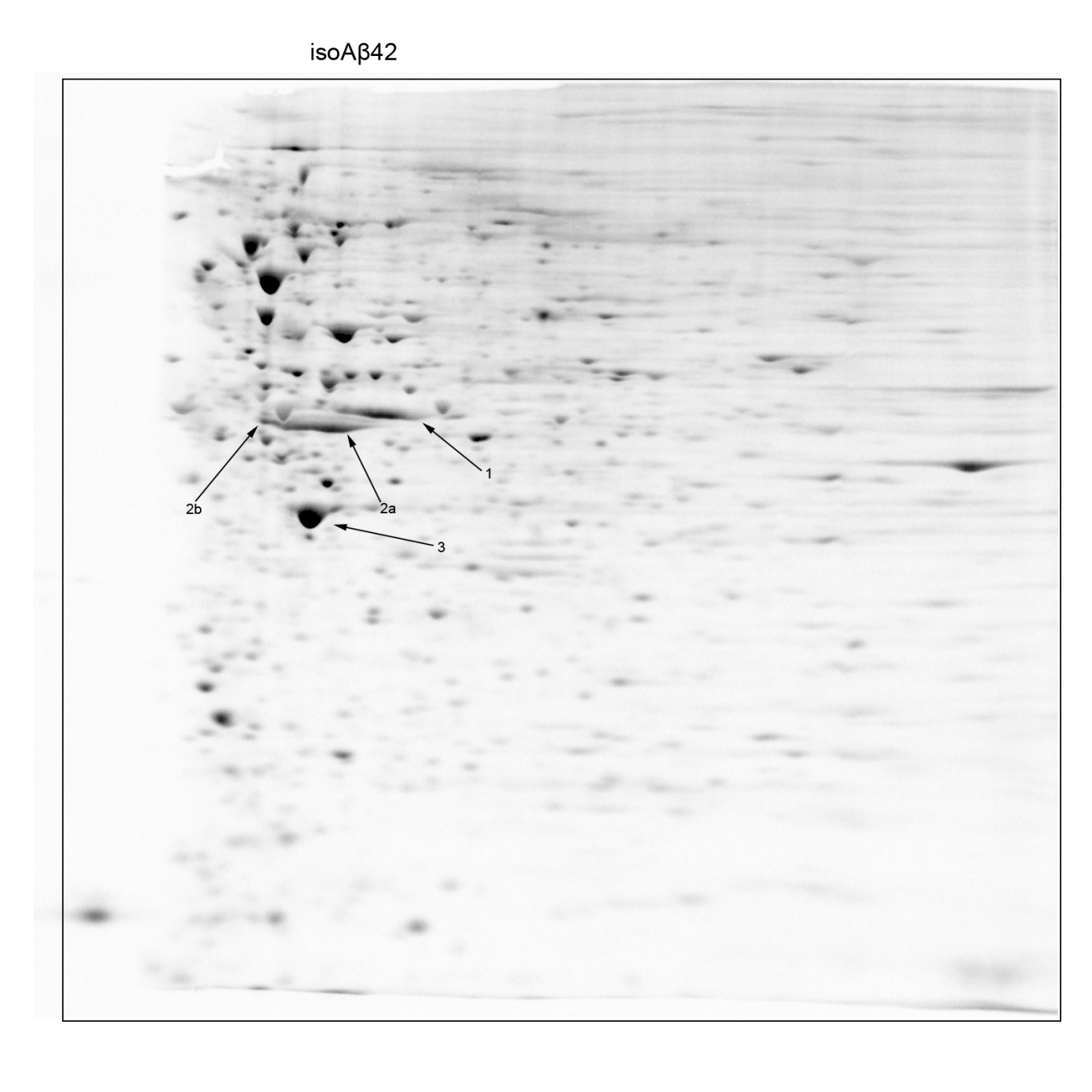


Western blot corresponding to Figure 4


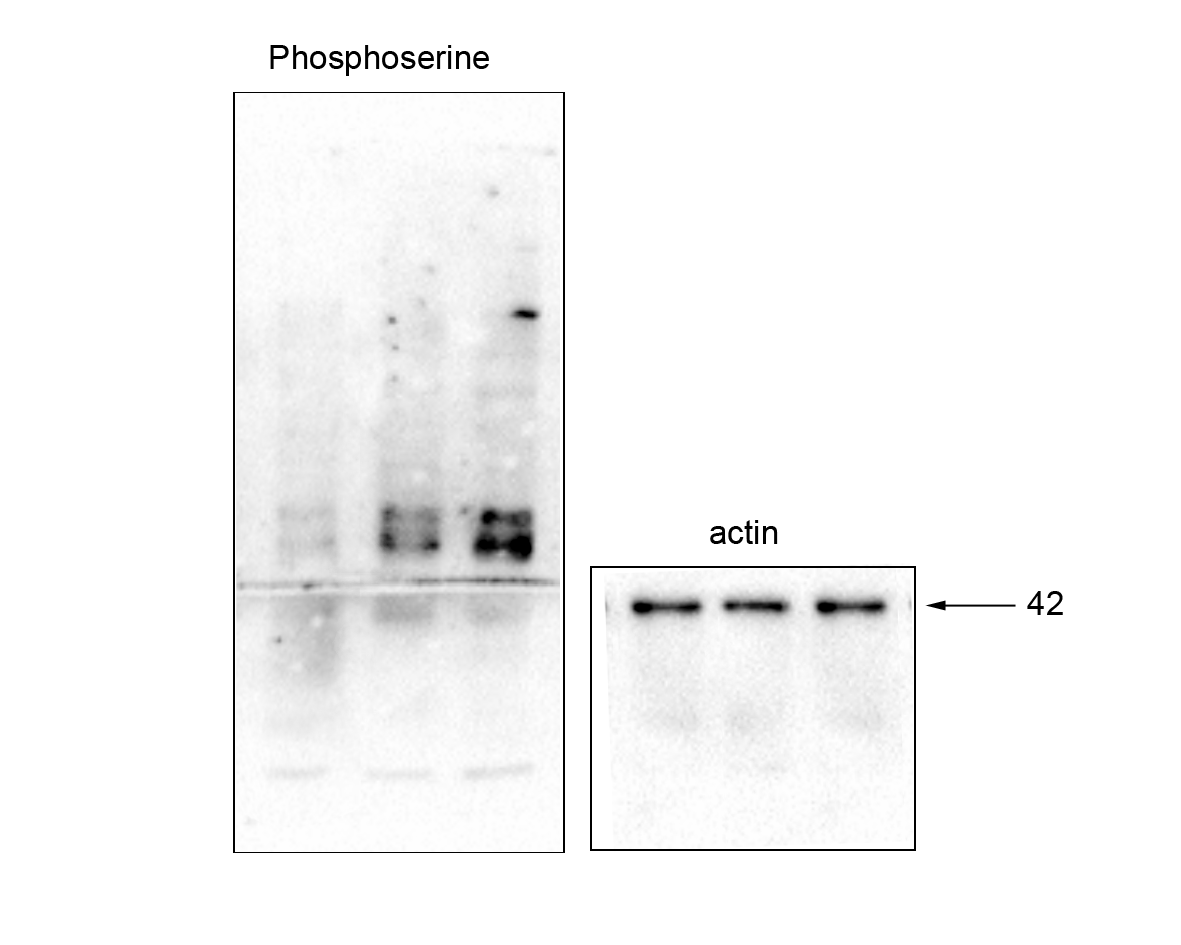


Ponso S staining for Western blots on Figure 5


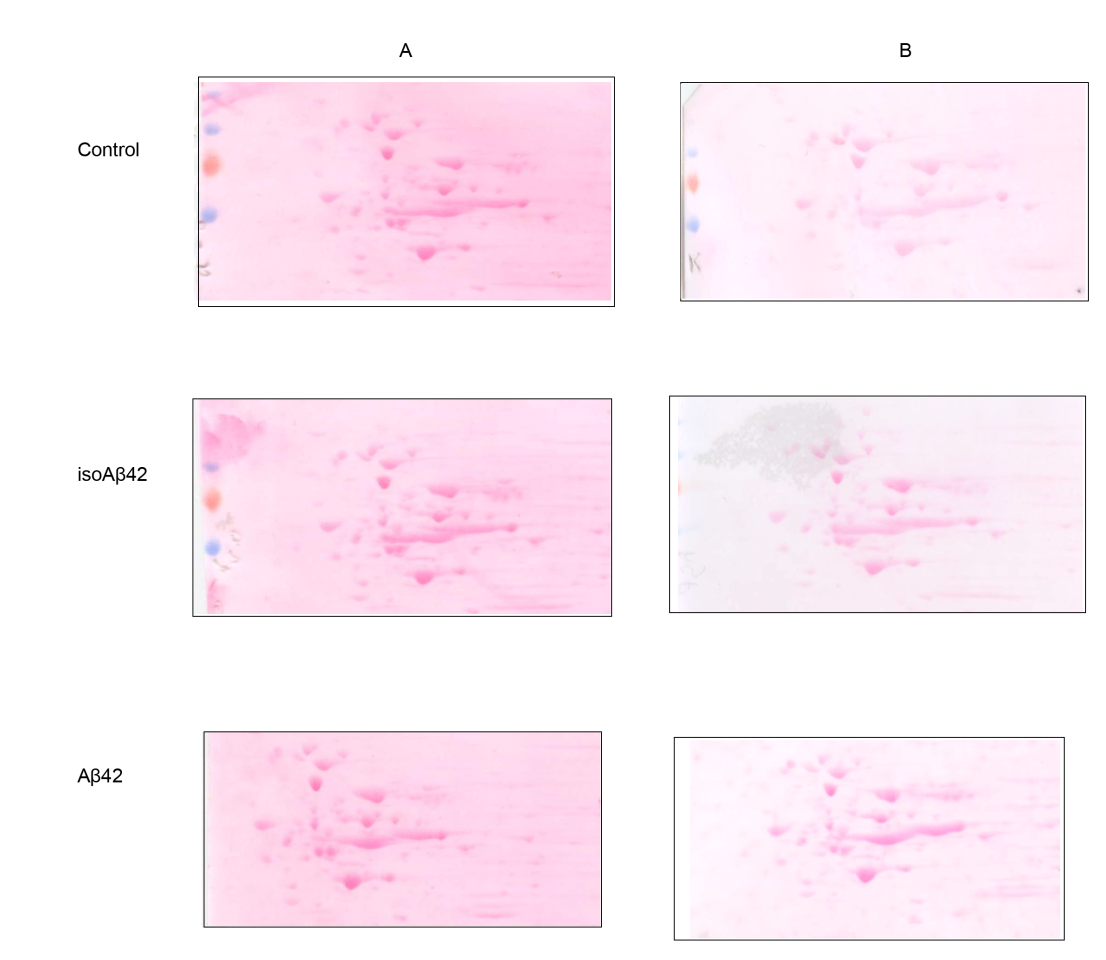


Full pictures for Figure 6. Visualization the changes in protein phosphorylation levels after SH-SY5Y cells incubation with Aβ42 and isoAβ42 (10 μM, 6 h). A-Coomassie staining , B- Pro-Q diamond phosphoprotein staining. 1 – α-tubulin, 2 - β-tubulin, 3- matrin 3, 4-cofilin-1. C- phosphorylated cofilin-1 stained with Pro-Q diamond phosphoprotein stain was normalized to its total amount stained with Coomassie R-250. Each value is the mean expressed as a percentage of the protein phosphorylation level in the control group ± SD of three independent experiments.


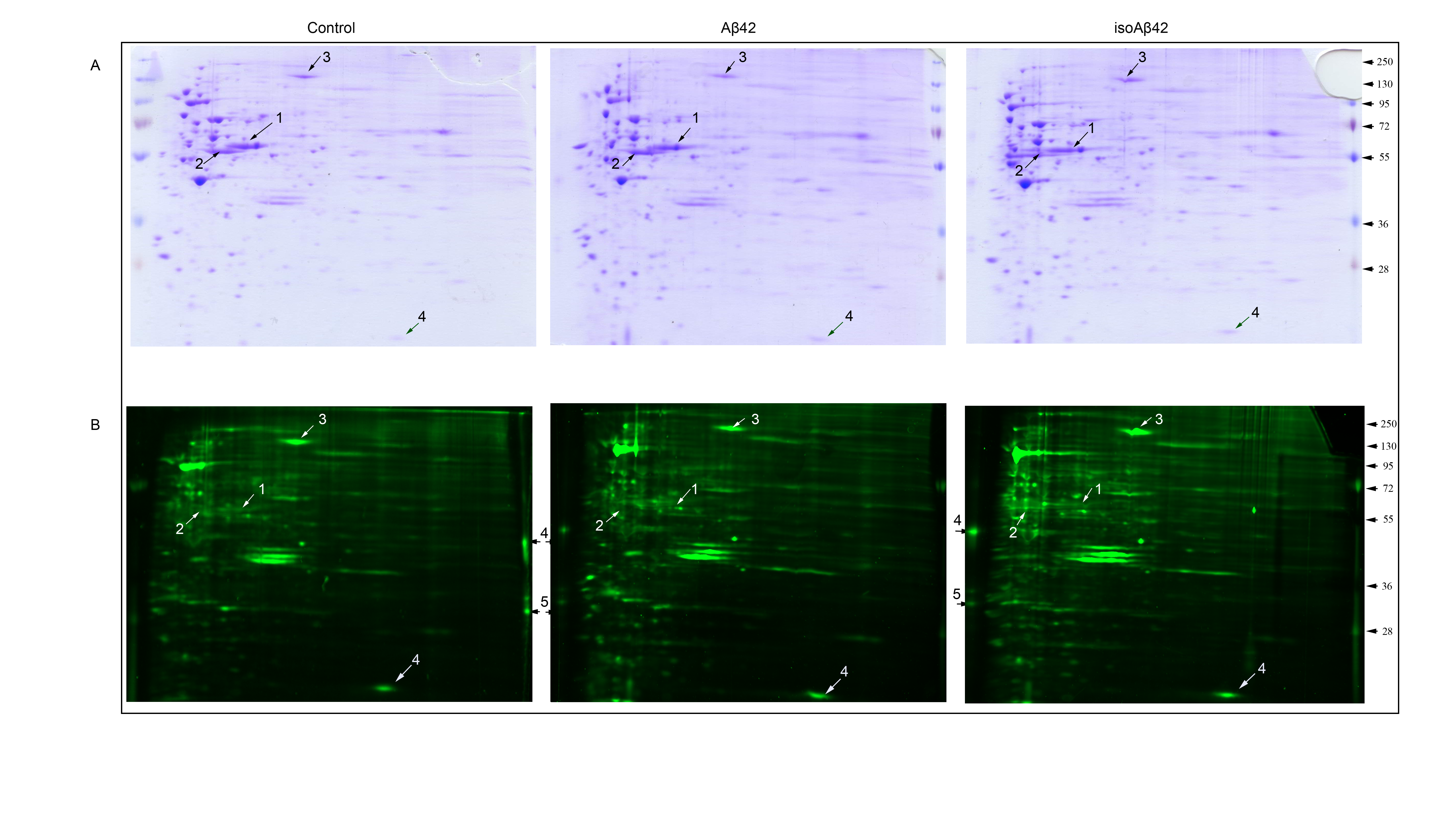


B

A


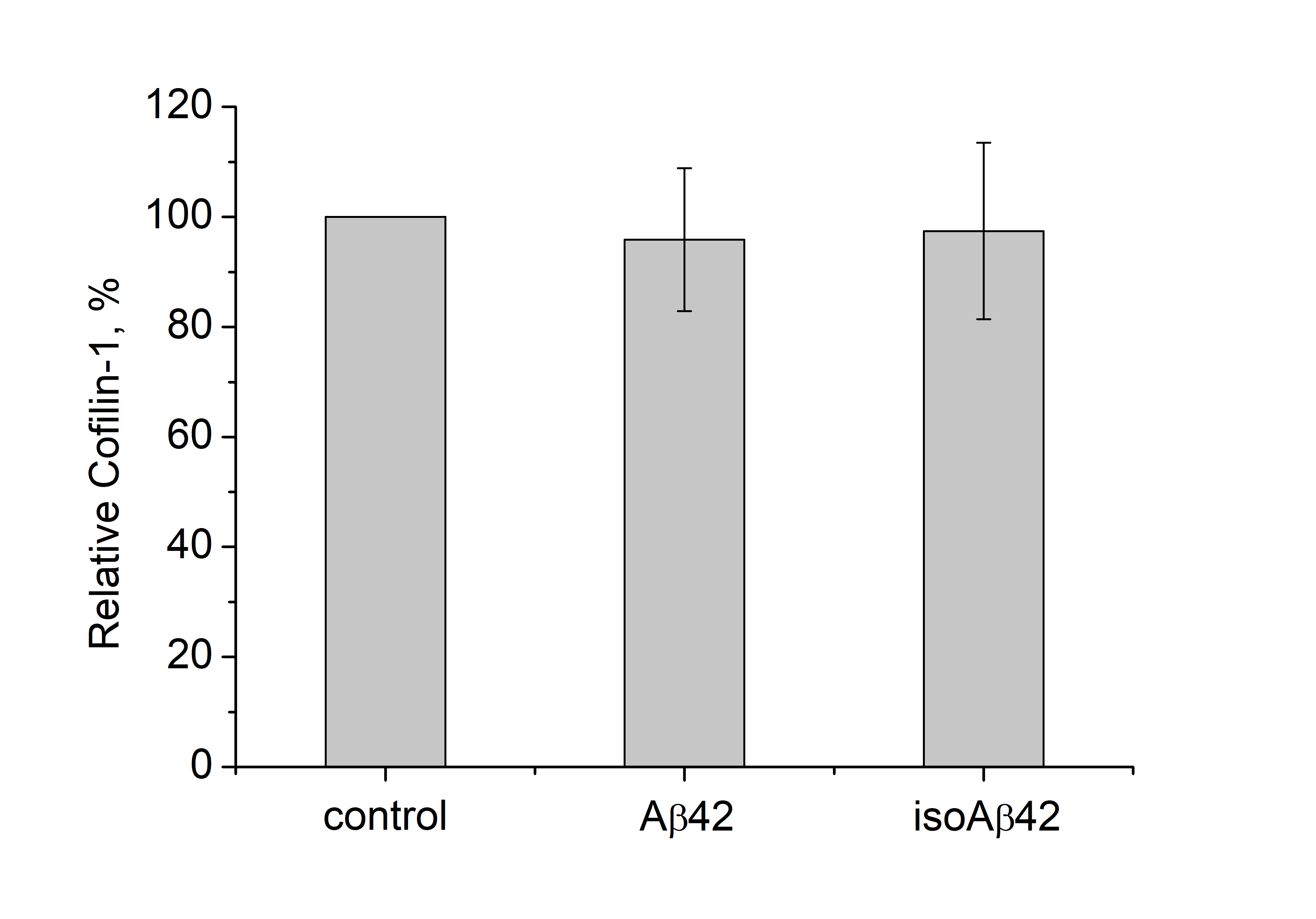


C

Western blots corresponding to Figure 8


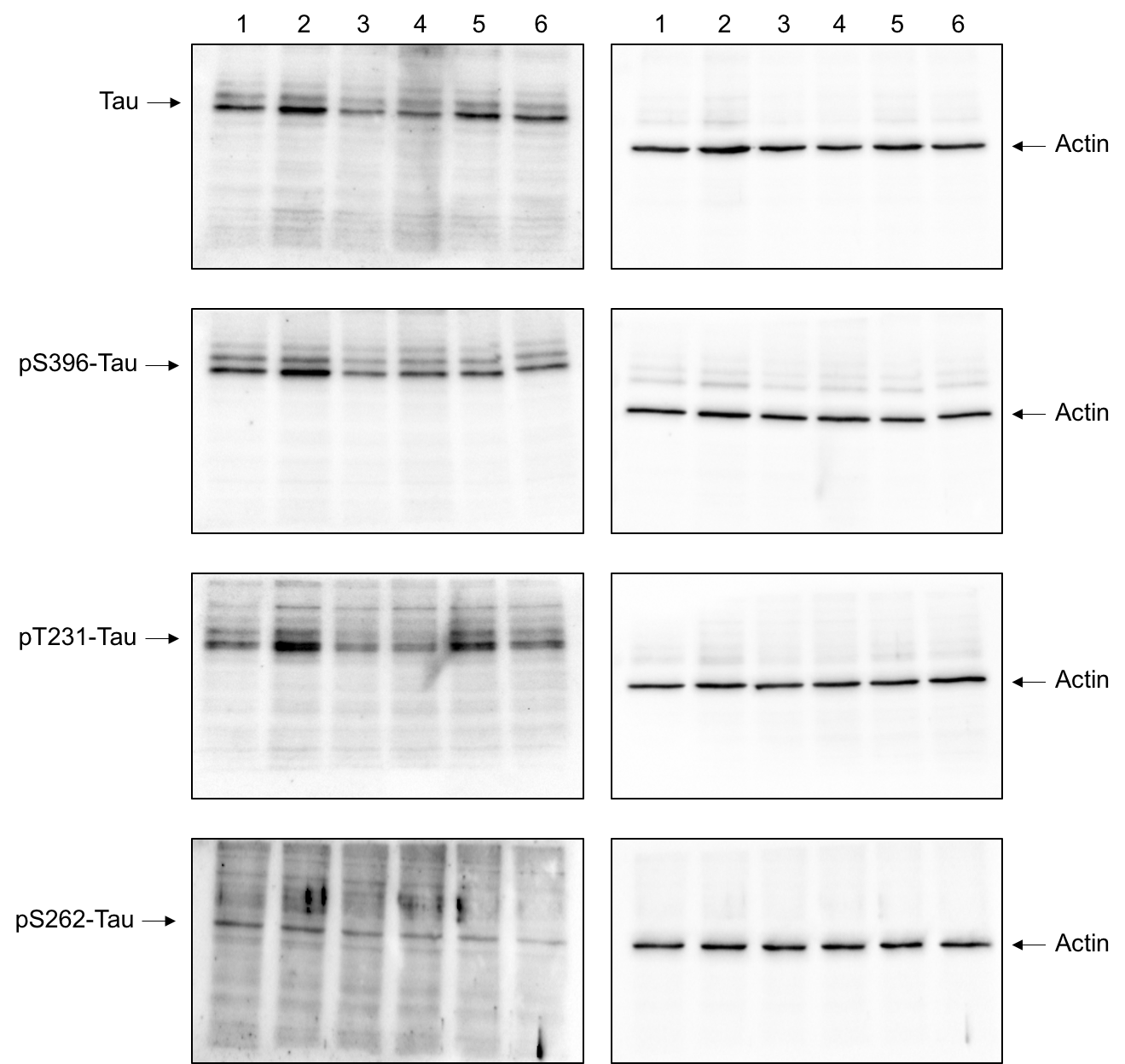

Supplement: Supplementary file 1 — Supplementary materials [file 41598_2018_21815_MOESM1_ESM.doc]
